# Somatostatin receptor expression for peptide receptor radionuclide therapy in Japanese patients with recurrent or metastatic differentiated thyroid cancer

**DOI:** 10.1007/s12149-026-02155-7

**Published:** 2026-01-23

**Authors:** Shiro Watanabe, Kenji Hirata, Junki Takenaka, Yamato Munakata, Keiichi Magota, Naoto Wakabayashi, Hiroto Koga, Kohsuke Kudo

**Affiliations:** 1 Department of Nuclear Medicine, Hokkaido University Hospital, Kita14, Nishi5, Kita-Ku, Sapporo, Hokkaido Japan; 2 Department of Diagnostic Imaging, Faculty of Medicine, Hokkaido University, Kita 15, Nishi 7, Kita-Ku, Sapporo, Hokkaido Japan; 3 Global Center for Biomedical Science and Engineering, Faculty of Medicine, Hokkaido University, Kita 15, Nishi 7, Kita-Ku, Sapporo, Hokkaido Japan; 4 Medical AI Research and Development Center, Hokkaido University Hospital, Kita14, Nishi5, Kita-Ku, Sapporo, Hokkaido Japan; 5 Healthcare AIX Innovation Center (HAIXIC), Hokkaido University, Kita 15, Nishi 7, Kita-Ku, Sapporo, Hokkaido Japan; 6 Comprehensive Heart Failure Center, University Hospital Würzburg, Am Schwarzenberg 15, Haus A15, 97078 Würzburg, Germany; 7 Division of Medical Imaging and Technology, Hokkaido University Hospital, Kita14, Nishi5, Kita-Ku, Sapporo, Hokkaido Japan; 8 Department of Diagnostic and Interventional Radiology, Hokkaido University Hospital, Kita 14, Nishi 5, Kita-Ku, Sapporo, Hokkaido Japan

**Keywords:** Differentiated thyroid carcinoma, Somatostatin receptor, Fluorodeoxyglucose, Radioiodine, Peptide receptor radionuclide therapy

## Abstract

**Objective:**

In this prospective study, we aimed to investigate the factors associated with somatostatin receptor (SSTR) expression in patients with recurrent or metastatic differentiated thyroid cancer (DTC).

**Methods:**

Patients with DTC scheduled for ^131^I therapy at the study institution were enrolled. The inclusion criteria required at least one assessable lesion (≥ 1 cm). SSTR expression was assessed by ^111^In-pentetreotide single-photon emission computed tomography/computed tomography (SPECT/CT), with Krenning scores of ≥ 2 considered positive. Fluorodeoxyglucose positron emission tomography (FDG PET) and post-^131^I therapy SPECT/CT were also evaluated. Quantitative evaluation was performed using standardized uptake values (SUV). Clinical factors were compared between SSTR-positive and SSTR-negative patients. Spearman’s correlation coefficient was calculated to evaluate the relationship between lesion size and SUVmax from ^111^In-pentetreotide SPECT, FDG PET, and ^131^I SPECT. Generalized estimating equations (GEEs) were utilized to identify predictors of SSTR positivity, accounting for within-patient correlation among multiple lesions. Furthermore, receiver operating characteristic (ROC) curves with clustered bootstrap resampling were used for evaluating the diagnostic performance of significant predictors.

**Results:**

Fourteen patients with DTC (six male; median age, 70.5 years) were evaluated, and 31 lesions were assessed. High SSTR expression was observed in 28.6% (4/14) of patients and 51.6% (16/31) of lesions. SSTR positivity was associated with follicular histology, elevated thyroglobulin (Tg) levels, ^131^I uptake, larger lesion size, and bone metastasis. Using GEEs accounting for intra-patient clustering, higher ^131^I SUVmax was the strongest independent predictor of SSTR positivity (*p* < 0.001). ROC analysis demonstrated ^131^I SUVmax yielded a high area under the curve of 0.93 (95% confidence interval: 0.65–1.00) for predicting SSTR positivity. SSTR SUVmax positively correlated with lesion size (ρ = 0.67), and ^131^I SUVmax (ρ = 0.73). Although ^18^F-FDG SUVmax moderately positively correlated with SSTR SUVmax (ρ = 0.49), it was not an independent predictor of SSTR positivity in GEE (*p* = 0.163).

**Conclusions:**

In this first study, high SSTR expression in Japanese patients with DTC was evaluated, demonstrating patients with follicular thyroid carcinoma, elevated Tg levels, larger tumor size, and positive ^131^I uptake are more likely to have SSTR-positive lesions. These findings might support the development of novel SSTR-targeted radiopharmaceutical therapies for DTC.

**Supplementary Information:**

The online version contains supplementary material available at 10.1007/s12149-026-02155-7.

## Introduction

Thyroid cancer is the most common type of endocrine cancer worldwide, particularly in East Asia [1]. Differentiated thyroid cancer (DTC), which includes papillary and follicular cancers, has been increasing in incidence in recent years [2]. However, most patients with DTC have good prognosis due to systemic treatment such as surgery and radioiodine (RAI) therapy. RAI therapy is commonly used as systemic therapy for recurrent or metastatic DTC. However, 5–22% of patients become refractory to standard therapy, characterized by low RAI uptake in metastatic lesions [3, 4]. Once the disease progresses to RAI-refractory DTC (RAIR-DTC), treatment options are limited to molecular-targeted tyrosine kinase inhibitors or therapies targeting genetic abnormalities [5]. Previous studies have shown that the average survival of patients with RAIR-DTC is approximately 2.5–3.5 years [6]. Therefore, there is a clear need for improved therapies for patients with DTC.

Peptide receptor radionuclide therapy (PRRT) has recently garnered attention as an effective treatment of neuroendocrine tumors (NETs) [7, 8]. ^177^Lu-DOTATATE is a PRRT agent that exerts its therapeutic effects through high binding affinity for somatostatin receptor (SSTR) subtype 2 (SSTR2) [9]. Somatostatins are a family of cyclopeptides produced by endocrine, gastrointestinal, neuronal, and immune cells [10]. Moreover, SSTR overexpression has been observed in patients with DTC [11, 12]. Although reported in a limited number of studies, a meta-analysis suggested that PRRT may also be useful for DTC [13]. If applicable to DTC, PRRT may represent a new treatment strategy for RAIR-DTC. Furthermore, PRRT may offer advantages such as avoiding the shortcomings of RAI therapy, including a low-iodine diet, long isolation periods, and salivary gland toxicity. Additionally, it may serve as an effective treatment option for iodine-responsive DTC. If cases of high SSTR expression are identified, eligible candidates for PRRT may be selected. In this study, we investigated the clinical factors associated with high SSTR expression in Japanese patients with recurrent or metastatic DTC to determine their eligibility for PRRT.

## Materials and methods

### Ethics approval and consent to participate

This prospective study was conducted in accordance with the principles of the Declaration of Helsinki. The study protocol was approved by the Ethics Committee of Hokkaido University and its Institutional Review Board (#SEI022-0032). According to the Ethical Guidelines for Medical Research on Human Subjects in Japan, the need for written consent was waived because the study did not involve any interventions or the use of human-derived samples, and patient anonymity was ensured. Verbal consent was obtained from all patients and documented in their electronic medical records.

### Patient enrollment

The study was conducted from October 2022 to March 2025. It included patients who were: (i) at least 20 years of age; (ii) pathologically diagnosed with DTC; (iii) confirmed to have at least one measurable lesion ≥ 1 cm on CT imaging; and (iv) scheduled to undergo RAI therapy. The exclusion criteria included patients who had: (i) difficulty undergoing scintigraphy imaging due to claustrophobia or other reasons; and (ii) poorly controlled diabetes. Patients with poorly controlled diabetes were excluded to avoid potential effects of hyperglycaemia on ^18^F-fluorodeoxyglucose (^18^F-FDG) uptake and quantitative accuracy, as the study prospectively analyzed standardized uptake values (SUVs) obtained from ^18^F-FDG positron emission tomography (PET).

### Imaging protocol

^111^In-pentetreotide scintigraphy was performed within 7 days before RAI administration to each patient. For ^111^In-pentetreotide, the administered activity was determined by pre- and post-injection syringe measurements. Whole-body planar images and single-photon emission computed tomography/computed tomography (SPECT/CT) of the target lesion were acquired 24 h after ^111^In-pentetreotide administration using a SPECT/CT system (Symbia Intevo Bold; Siemens Healthineers, Erlangen, Germany). ^111^In-pentetreotide SPECT scans were performed using medium-energy low-penetration collimators, photopeaks at 172 and 245 keV with 15% width, 60 views (30 per detector) in step-and-shoot mode with auto-contouring, and a 128 × 128 matrix (zoom = 1) with a 2.36 mm pixel size. Additionally, CT was performed with a 130 kV voltage, 80 mAs quality reference, 1.2 pitch, 0.6 s rotation time, and 3.0 mm slice thickness. Reconstruction for attenuation correction was performed using the B08 filter. The SPECT iterative reconstruction protocol was based on the Siemens ordered-subset conjugate-gradient method with 20 iterations and two subsets, without scatter correction.

All patients received their first RAI administration with a fixed activity of 5.55 GBq. No patient underwent repeated or multiple RAI therapy, as this study evaluated sodium/iodide symporter (NIS) expression based on post-therapeutic scintigraphy findings. Pretreatment preparation included iodine restriction for at least 3 weeks and either levothyroxine withdrawal for 3 weeks or recombinant human thyroid-stimulating hormone (rhTSH) administration, targeting a thyroid-stimulating hormone (TSH) level ≥ 30 µIU/mL at the time of treatment. If TSH levels did not reach ≥ 30 µIU/mL after levothyroxine discontinuation, rhTSH was administered. The administered dose of ^131^I was fixed at 5.55 GBq. Post-therapy ^131^I scintigraphy, including whole-body planar imaging and SPECT/CT, was performed 72 h after RAI administration using the same SPECT/CT system. If isolation exceeded 72 h, imaging was performed on the day the patient was released. ^131^I SPECT scans were performed using high-energy collimators, a photopeak at 364 keV with 20% width, 60 views (30 per detector) in step-and-shoot mode with auto-contouring, and a 128 × 128 matrix (zoom = 1) with a 2.36 mm pixel size. The SPECT iterative reconstruction protocol was developed using the same method as the ^111^In-pentetreotide SPECT.

Additionally, ^18^F-FDG PET/CT scans performed within 180 days prior to the study were included in the analysis. As part of the clinical protocol, all ^18^F-FDG PET/CT scans were acquired and reconstructed in accordance with the institutional standards, following the 2018 guidelines established by the Japanese Society of Nuclear Medicine. Before tracer injection, the patient fasted for at least 6 h. Following a blood glucose test to confirm blood glucose levels < 150 mg/dL, PET images were acquired 60 min after an intravenous injection of ^18^F-FDG. Regarding ^18^F-FDG, doses were administered using an automatic injector; the actual administered activity automatically recorded by the system was used.

### Imaging analysis

Image interpretation was performed independently by a single board-certified nuclear medicine physician with 14 years of experience. Considering image analysis, all lesions ≥ 1 cm were evaluated. Lesion size was determined based on CT images obtained during ^111^In-pentetreotide SPECT/CT. In accordance with the Response Evaluation Criteria in Solid Tumors version 1.1 guidelines, the longest diameter was measured for general lesions and the short axis for lymph node metastases. Bone lesions were assessed only when an associated soft-tissue component was present. SPECT images were not used for morphological measurements. A qualitative analysis of ^111^In-pentetreotide scintigraphy was performed based on the Krenning score (KS) [14]. As physiological uptake in post-therapy ^131^I scintigraphy varies among individuals, lesion uptake was graded based on relative intensity as follows: 1 = uptake equal to or lower than background activity; 2 = uptake higher than background but not exceeding all normal organs except the thyroid bed; and 3 = uptake greater than all normal organs except the thyroid bed. Lesions with a score of 2 or higher were considered positive. ^18^F-FDG PET/CT positivity was determined according to the Deauville criteria, with scores ≥ 3 considered positive [15]. Additionally, In regard to each scintigraphy and ^18^F-FDG PET scan, the maximum standardized uptake value (SUVmax) was measured for each lesion and recorded as SSTR SUVmax, ^131^I SUVmax, and ^18^F-FDG SUVmax. Quantitative analysis was performed using SUV measurements; however, the quantitative accuracy of SUV using SPECT has already been confirmed [16]. To ensure the reliability of our quantitative data, cross-calibration between the SPECT/CT system and the dose calibrator sensitivity was performed monthly for each specific combination of radionuclide and collimator used in the study. Additionally, reconstruction parameters were kept constant throughout the study period to eliminate variability in SUV values that could result from parameter changes.

### Statistical analysis

All values are expressed as medians and ranges. Patients and lesions were allocated into two groups based on SSTR positivity. Cases of at least one SSTR-positive lesion were defined as SSTR-positive, whereas all other cases were defined as SSTR-negative. Similarly, cases of at least one positive lesion on ^131^I and ^18^F-FDG imaging were defined as positive. To avoid bias from patients with multiple lesions, a representative lesion analysis was conducted using up to five of the largest lesions per patient, with a maximum of two lesions per organ.

All data were analyzed using JMP^®^ Version 17.0 (SAS Institute Inc., Cary, NC, 1989–2024) and R software (version 4.5.1; R Foundation for Statistical Computing, Vienna, Austria). Differences in parameters between the two groups were evaluated by the Wilcoxon rank-sum test and Fisher’s exact test, as appropriate. Spearman’s correlation coefficient was used for evaluating the relationship between lesion size and SSTR SUVmax, ^131^I SUVmax, and ^18^F-FDG SUVmax. As multiple lesions were nested within individual patients, stratified bootstrap resampling (2,000 replicates, stratified by patient ID) was performed to obtain bias-corrected standard errors and 95% confidence intervals (95% CI) using the percentile method. Univariate logistic regression was used for assessing clinical and imaging predictors of SSTR positivity. The primary analysis to identify predictors of SSTR positivity was performed using generalized estimating equations (GEEs) with a logit link, specifying an exchangeable working correlation structure to account for within-patient correlation. To address the numerical instability observed in the initial model iterations, all continuous predictor variables were standardized to z-scores prior to model fitting. Multicollinearity was assessed by the Variance Inflation Factor (VIF); all VIF values were within acceptable limits (VIF < 2.0). Odds ratios (ORs) are reported per one standard deviation (SD) increase in the predictor variable. As sensitivity analyses, generalized linear mixed-effects models (GLMMs) and Firth’s penalized logistic regression were applied to assess the robustness of the findings. Furthermore, the diagnostic performance of significant predictive factors was evaluated by receiver operating characteristic (ROC) curves. The area under the curve (AUC) was calculated for each predictor. The 95%CIs for the AUCs were derived using a patient-level clustered bootstrap method (2,000 resamples). Furthermore, the clustered bootstrap distribution was used for estimating the distribution of the optimal cutoff value determined by maximizing the Youden Index. The uncertainty in the optimal cutoff was reported as the median and interquartile range (IQR) of its bootstrap distribution. Statistical significance was set at *p* < 0.05. Given the exploratory nature and relatively small sample size of this study, no formal adjustment for multiplicity was applied. The results should be interpreted as hypothesis-generating.

## Results

### Patients’ characteristics

Seventeen patients were initially enrolled in this study. Three patients were excluded: one due to an acute exacerbation before ^131^I administration resulting in discontinued treatment; another due to unavailable pentetreotide data; and the third patient, after total thyroidectomy and ^131^I treatment, was diagnosed with kidney cancer rather than thyroid cancer based on histopathological examination of resected lung tissue.

Consequently, 14 patients (six male and eight female participants) were evaluated (Table 1), with a median age of 70.5 years (range: 50–83 years). Histological subtypes included papillary carcinoma in eleven patients and follicular carcinoma in three patients. A total of 31 evaluable lesions were identified (Table 2), with a median lesion size of 16.9 mm (range: 10.8–50.8 mm). Of these, there were two local recurrences, nine lymph node metastases, three pulmonary metastases, and seventeen bone metastases. Post-therapy ^131^I scintigraphy was performed 72 h after RAI administration in 12 patients, 96 h in 1 patient, and 10 days (240 h) in 1 patient. Regarding the representative lesion analysis (*n* = 19) (Table 2), the median lesion size was 20.0 mm (range: 11.1–50.8 mm). There were two cases of local recurrence, seven cases of lymph node metastasis, three cases of lung metastasis, and seven cases of bone metastasis. The median and range of lesion sizes by organ for both all evaluable and representative lesions are summarized in Supplementary Tables S1 and S2. The median dose of ^111^In-pentetreotide was 191.3 MBq (range: 164.3–216.6 MBq), and the median dose of ^18^F-FDG per body weight was 4.3 MBq/kg (range: 2.4–4.6 MBq/kg). The median ^18^F-FDG uptake time was 59 min (range: 50–67 min), and the median blood glucose was 106 mg/dL (range: 90–128 mg/dL).

**Table 1:** Patients’ details and comparison of SSTR expression

Parameter		All patients (*n* = 14)	(%)	SSTR + (*n* = 4)	SSTR - (*n* = 10)	*p* value
Age (year, median (range))		70.5 (50–83)		67.5 (50–73)	71.0 (54–83)	0.227
Gender (n)						1.000
	Female	8	57.1	2	6	
	Male	6	42.9	2	4	
Body weight (kg, median (range))		61.5 (43.8–94.0)		65.5 (52.0–94.0)	60.0 (44.0–102.0)	0.437
Pathology (n)						*0.011
	Papillary	11	78.6	**1**	**10**	
	Follicular	3	21.4	**3**	**0**	
TSH elevation (n)						0.239
	Levothyroxine withdrawal	9	64.3	2	7	
	rhTSH	2	14.3	0	2	
	Combined both	3	21.4	2	1	
TSH (µIU/mL, median (range))		92.5 (19.6–373.5)		120.8 (19.6–276.65)	92.5 (25.0–373.5)	0.724
Tg (µg/mL, median (range))		941 (0–184230)		**44,366** **(1490–184230)**	**299** **(0–1035.0)**	*0.006
Tg antibody (n)						1.000
	≥ 20	2	14.3	0	2	
	< 20	12	85.7	4	8	
FDG uptake (n)						1.000
	Positive	12	85.7	4	8	
	Negative	2	14.3	0	2	
^131^I uptake (n)						*0.041
	Positive	4	28.6	**3**	**1**	
	Negative	10	71.4	**1**	**9**	

FDG, fluorodeoxyglucose; rhTSH, recombinant human thyroid-stimulating hormone; SSTR, somatostatin receptor; Tg, thyroglobulin; TSH, thyroid-stimulating hormone

**Table 2:** Lesions’ characteristics

Parameter		All lesions (*n* = 31)		Representative-lesions (*n* = 19)	
		n	(%)	n	(%)
Number per patient (n)		1 (1–12)		1 (1–2)	
Size (mm, median (range))		16.9 (10.8–50.8)		20.0 (11.1–50.8)	
Location					
	Lymph node	9	29.0	7	36.8
	Lung	3	9.7	3	15.8
	Bone	17	54.8	7	36.8
	Local recurrence	2	6.5	2	10.5
FDG score					
	1	1	3.2	1	5.3
	2	2	6.5	2	10.5
	3	2	6.5	2	10.5
	4	9	29.0	3	15.8
	5	17	54.8	11	57.9
FDG SUVmax (median (range))		8.3 (1.0–17.7)		8.3 (1.0–17.7)	
^131^I score					
	1	13	41.9	11	57.9
	2	4	12.9	1	5.3
	3	14	45.2	7	36.8
^131^I SUVmax (median (range))		1.3 (0.0–54.4)		0.1 (0.0–43.6)	
SSTR score					
	0	5	16.1	5	26.3
	1	11	35.5	7	36.8
	2	5	16.1	2	10.5
	3	9	29.0	4	21.1
	4	1	3.2	1	5.3
SSTR SUVmax (median (range))		0.8 (0.1–7.0)		0.5 (0.1–7.0)	

FDG, fluorodeoxyglucose; SSTR, somatostatin receptor; SUV, standardized uptake value

### SSTR expression and predictive factors

Four out of fourteen patients (28.6%) tested positive for SSTR expression (Table 1). SSTR-positive cases were significantly more likely to have follicular carcinoma (*p* = 0.011), high thyroglobulin (Tg) levels (*p* = 0.006), and positive ^131^I uptake (*p* = 0.04) compared with SSTR-negative cases. Notably, all three cases (100%) of follicular carcinoma were positive for SSTR expression. There was no significant difference in the frequency of positive ^18^F-FDG uptake (*p* = 1.000).

**Fig. 1:**
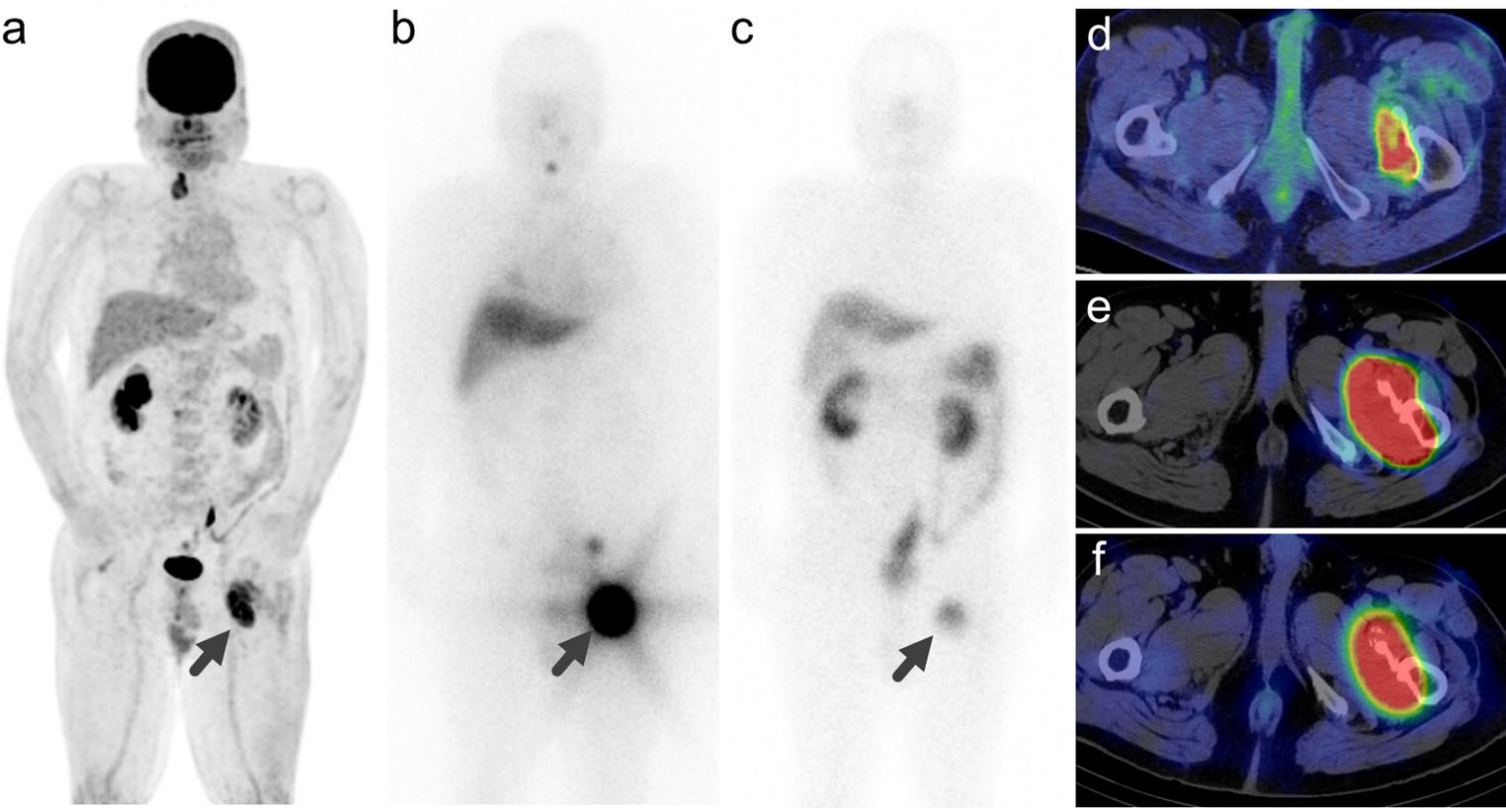
Representative case 1. A case of a man in his 60s with follicular thyroid carcinoma and metastasis to the left femur. (**a**) Maximum intensity projection of ^18^F-FDG PET showing increased uptake corresponding to the left femoral metastasis (Score 5/5, SUVmax = 9.1). (**b**) Post-treatment ^131^I scintigraphy performed at 10 days after ^131^I administration demonstrated significant iodine uptake (Score 3/3, SUVmax = 33.7). (**c**)^111^In-pentetreotide scintigraphy showing intense uptake, stronger than that in the liver (Score 3/4, SUVmax = 5.4). (**d**) Fused image of ^18^F-FDG PET and CT. (**e**) Fused image of ^131^I SPECT and CT. (**f**) Fused image of ^111^In-pentetreotide SPECT and CT

**Fig. 2:**
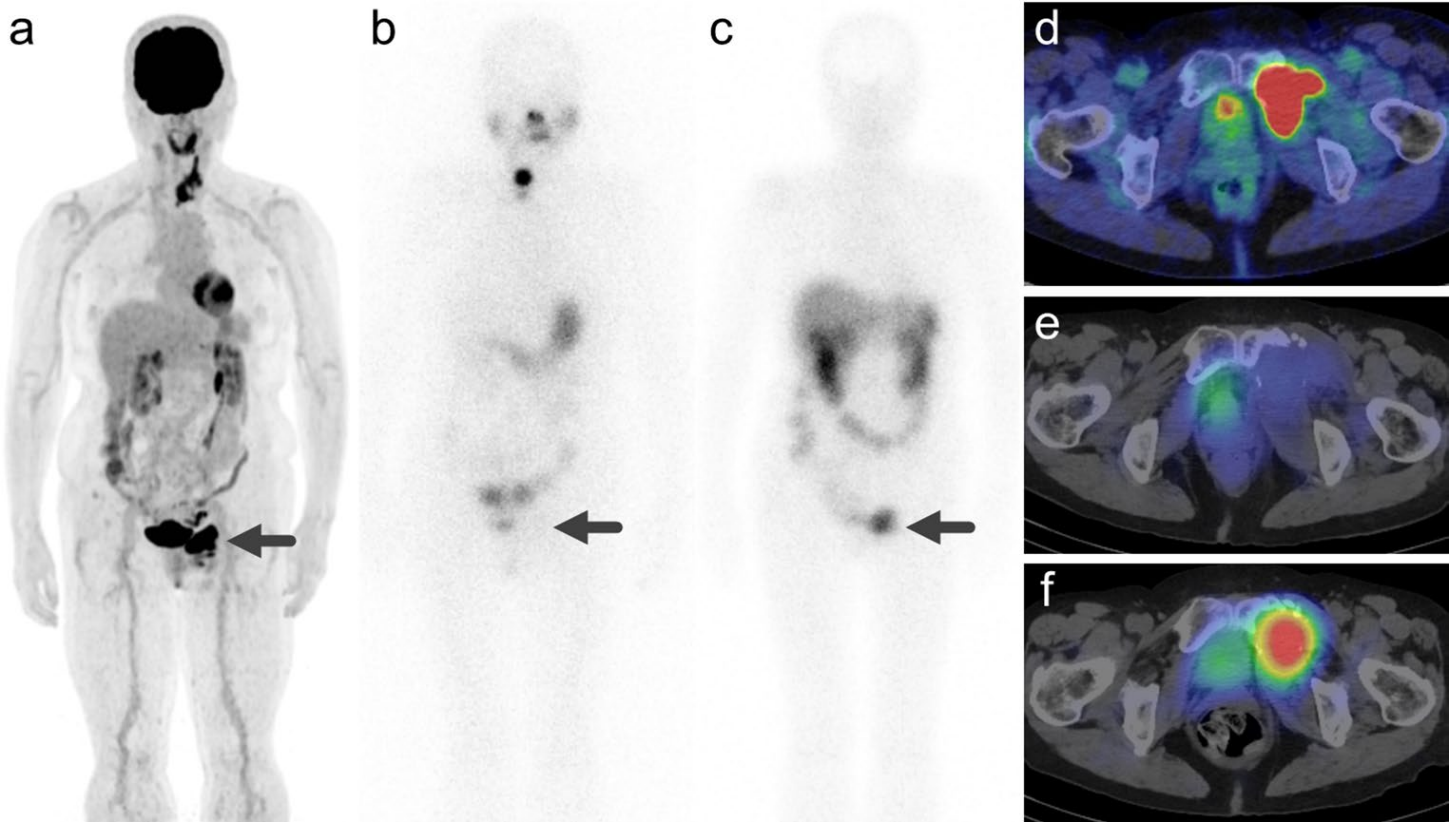
Representative case 2. A case of a woman in her 70s with papillary thyroid carcinoma and metastasis to the left pubic bone. (**a**) Maximum intensity projection of ^18^F-FDG PET showing increased uptake corresponding to the left pubic bone metastasis (Score 5/5, SUVmax = 12.2). (**b**) Post-treatment ^131^I scintigraphy performed at 3 days after ^131^I administration revealed no significant iodine uptake (Score 1/3, SUVmax = 0.04). (**c**) ^111^In-pentetreotide scintigraphy showing intense uptake, stronger than that in the liver, was observed (Score 3/4, SUVmax = 5.0). (**d**) Fused image of ^18^F-FDG PET and CT. (**e**) Fused image of ^131^I SPECT and CT. (**f**) Fused image of ^111^In-pentetreotide SPECT and CT

Of the 31 lesions, 16 (51.6%) were positive for SSTR expression (Table 3). SSTR-positive lesions were significantly larger (*p* = 0.015), more likely to be bone metastases (*p* < 0.001), had higher ^18^F-FDG SUVmax (*p* = 0.023), were positive for ^131^I uptake (*p* < 0.001), and had higher ^131^I SUVmax (*p* < 0.001). No significant differences were observed in the frequency of ^18^F-FDG uptake (*p* = 0.101). Of the nineteen representative lesions, seven (36.8%) were SSTR-positive (Table 3). These lesions were significantly larger (*p* < 0.001), more likely to be bone metastases (*p* = 0.007), positive for ^131^I uptake (*p* = 0.006), and had higher ^131^I SUVmax (*p* = 0.006). There were no significant differences in the frequency of positive ^18^F-FDG uptake (*p* = 0.263) or ^18^F-FDG SUVmax (*p* = 0.220). Univariate logistic regression analysis of all lesions (Table 4) revealed that lesion size and ^131^I SUVmax were predictive factors for SSTR positivity (*p* = 0.020 and 0.007, respectively). Univariate logistic regression analysis of the representative lesions (Table 4) revealed that only ^131^I SUVmax was a predictive factor for SSTR positivity (*p* = 0.037), whereas lesion size did not reach statistical significance (*p* = 0.09). Representative cases are shown in Figs. 1 and 2.

**Table 3:** Comparison of all lesions (*n* = 31) and the Representative-lesions (*n* = 19) in SSTR expression

Parameters		All lesions (*n* = 31)			Representative-lesions (*n* = 19)		
		SSTR-positive (*n* = 16)	SSTR-negative (*n* = 15)	p value	SSTR-positive (*n* = 7)	SSTR-negative (*n* = 12)	p value
Size (mm, median (range))		**28.8** **(10.8–50.8)**	**15.6** **(11.1–24.9)**	**0.015**	**41.0** **(20.1–50.8)**	**15.9** **(11.1–24.9)**	*<0.001
Location (n)				*<0.001			*0.007
	Lymph node	**1**	**8**		**1**	**6**	
	Lung	**0**	**3**		**0**	**3**	
	Bone	**15**	**2**		**6**	**1**	
	Trachea	**0**	**2**		**0**	**2**	
^18^F-FDG uptake (n)				0.101			0.263
	Positive	16	12		7	9	
	Negative	0	3		0	3	
^18^F-FDG SUVmax (median (range))		**8.9** **(4.7–12.2)**	**6.0** **(0.9–17.7)**	*0.023	8.7 (4.7–12.2)	6.7 (1.0–17.7)	0.220
^131^I uptake (n)				*<0.001			*0.006
	Positive	**15**	**3**		**6**	**2**	
	Negative	**1**	**12**		**1**	**6**	
^131^I SUVmax (median (range))		**10.3** **(0.0–54.4)**	**0.1** **(0.0–9.0)**	*<0.001	**22.5** **(0.0–43.6)**	**0.1** **(0.0–9.0)**	*0.006

Note: SSTR-positive lesions generally exhibited larger sizes and higher ^131^I SUVmax compared to SSTR-negative lesions, whereas ^18^F-FDG SUVmax showed overlapping distributions between the two groups

FDG, fluorodeoxyglucose; SSTR, somatostatin receptor; SUV, standardized uptake value

Interpretive note: The SSTR-positive lesions, particularly bone metastases, demonstrated the largest median size and highest ^131^I SUVmax, underscoring the strong association between SSTR expression, lesion size, and radioiodine avidity across different metastatic sites

**Table 4:** Univariate logistic regression analysis of SSTR-positive lesions

	Parameter	Odds ratio	[95% CI]	*p*
All lesions (*n* = 31)	**Size**	**1.16**	**1.05–1.27**	*0.020
	^18^F-FDG SUVmax	1.18	0.97–1.52	0.132
	^**131**^ **I SUVmax**	**1.65**	**1.14–2.37**	*0.007
Representative-lesions (*n* = 19)	Size	1.4	0.95–2.05	0.086
	^18^F-FDG SUVmax	1.07	0.87–1.33	0.518
	^**131**^ **I SUVmax**	**1.4**	**1.02–1.92**	*0.037

CI, confidence interval; FDG, fluorodeoxyglucose; SSTR, somatostatin receptor; SUV, standardized uptake value

Table 5 summarizes the relationships between imaging parameters and SSTR positivity. Regarding the primary analysis using GEE models accounting for intra-patient clustering, higher ^131^I SUVmax was significantly associated with SSTR positivity (OR, 35.09; 95%CI, 7.08–173.77, *p* < 0.001), whereas ^18^F-FDG SUVmax (OR, 1.63; 95%CI, 0.82–3.26, *p* = 0.163) and lesion size (OR, 0.65; 95%CI, 0.43–3.88, *p* = 0.649) showed no significant association. The sensitivity analyses yielded consistent results; notably, in the GLMMs with random intercepts for patients, ^131^I SUVmax tended to be positively associated with SSTR positivity (OR, 6.00, *p* = 0.072), while lesion size also showed a trend toward significance (OR, 1.49, *p* = 0.065). ^18^F-FDG SUVmax was not associated with SSTR positivity (*p* = 0.64). Firth-corrected logistic regression confirmed the robustness of the associations for ^131^I SUVmax and lesion size. ^131^I SUVmax remained significantly associated with SSTR positivity (OR, 1.51; 95% CI: 1.17–5.27, *p* < 0.001), as did lesion size (OR, 1.13; 95% CI: 1.04–1.31, *p* = 0.002), further supporting the primary GEE results. ^18^F-FDG SUVmax did not reach statistical significance (*p* = 0.13). Collectively, these results suggest that both ^131^I avidity and lesion size are potential predictors of SSTR expression. Although ^18^F-FDG SUVmax showed a moderate positive correlation with SSTR SUVmax, it did not emerge as an independent predictor.

**Table 5:** Association between imaging parameters and SSTR positivity

Predictor	Model type	Odds ratio	95% CI	*p*-value	Interpretation
^131^I-SUV	GEE (exchangeable)*	35.09**	—	< 0.001	Significant positive association
^131^I-SUV	Mixed-effects logistic regression	6.00	—	0.072	Trend toward significance
^131^I-SUV	Firth penalized logistic regression	1.51	1.17–5.27	< 0.001	Significant positive association
^18^F-FDG SUVmax	GEE (exchangeable)*	1.63**	—	0.163	No association
^18^F-FDG SUVmax	Mixed-effects logistic regression	8.08	—	0.64	No association
^18^F-FDG SUVmax	Firth penalized logistic regression	1.16	0.96–1.46	0.13	No association
Lesion size (mm)	GEE (exchangeable)*	1.29**	—	0.649	No association
Lesion size (mm)	Mixed-effects logistic regression	1.49	—	0.065	Trend toward significance
Lesion size (mm)	Firth penalized logistic regression	1.13	1.04–1.31	0.002	Significant positive association

FDG, fluorodeoxyglucose; GEE, generalized estimating equation; SUV, standardized uptake value; SSTR, somatostatin receptor

*In GEE, all continuous predictor variables were standardized to z-scores prior to model fitting

**Odds ratios are reported per one standard deviation increase in each standardized predictor variable

**Fig. 3:**
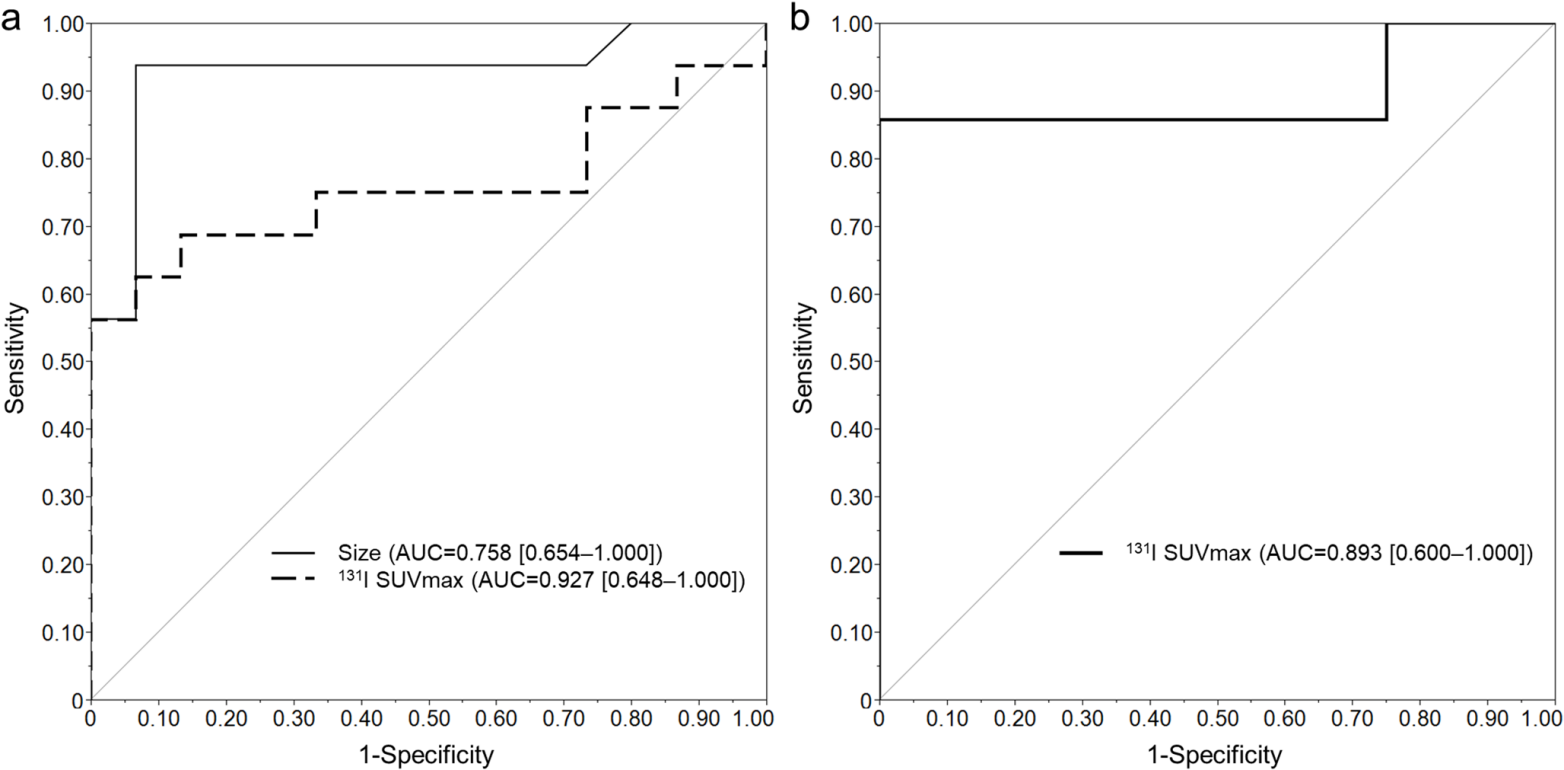
Results of ROC analysis. (**a**) Analysis of all lesions for SSTR expression using lesion size and ^131^I SUVmax. Lesion size showed the AUC of 0.76 (95% CI; 0.65–1.00), and ^131^I SUVmax demonstrated the AUC of 0.93 (95% CI; 0.65–1.00). (**b**) Representative lesions analyzed for SSTR expression using ^131^I SUVmax. ^131^I SUVmax showed an AUC of 0.89 (95% CI; 0.60–1.00)

ROC analysis of all SSTR-positive lesions, based on clustered bootstrap resampling, the optimal cutoff for lesion size and ^131^I SUVmax was 21.6 (median; IQR: 20.1–25.9) and 0.92 (median; IQR: 0.92–5.0). ^131^I SUVmax and lesion size showed AUCs of 0.76 (95% CI: 0.65–1.00) and 0.93 (95% CI: 0.65–1.00), respectively (Fig. 3a). In the representative lesions, the bootstrapped optimal cutoff value for ^131^I SUVmax had a median of 9.4 (IQR: 5.0–11.34). The AUC was 0.89 for ^131^I SUVmax (95% CI: 0.60–1.00) (Fig. 3b).

### Relationship between SSTR expression and ^18^F-FDG / ^131^I uptake

**Fig. 4:**
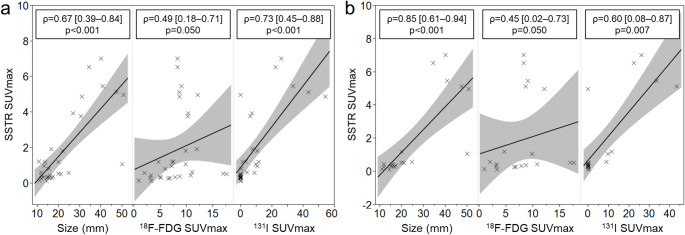
Scatterplots and correlations in (**a**) all lesions and (**b**) representative lesions analysis between SSTR SUVmax and size, ^18^F-FDG /^131^I SUVmax. The relationship was evaluated by Spearman’s correlation coefficient (ρ)

Quantitative analysis of all lesions revealed a significant positive correlation between SSTR SUVmax and lesion size (ρ = 0.67; 95% CI, 0.39–0.84; *p* < 0.001), ^18^F-FDG SUVmax (ρ = 0.49; 95% CI, 0.18–0.71; *p* = 0.050), and ^131^I SUVmax (ρ = 0.73; 95% CI, 0.45–0.88; *p* < 0.001) (Fig. 4a). Similarly, analysis of representative lesions showed a significant positive correlation between SSTR SUVmax and lesion size (ρ = 0.85; 95% CI, 0.61–0.94; *p* < 0.001), ^18^F-FDG SUVmax (ρ = 0.45; 95% CI, 0.02–0.73; *p* = 0.050), and ^131^I SUVmax (ρ = 0.60; 95% CI, 0.08–0.87; *p* = 0.007) (Fig. 4b).

## Discussion

This prospective study investigated the factors associated with high SSTR expression in Japanese patients with recurrent or metastatic DTC. In our cohort, at the patient level, high SSTR positivity was more frequently observed in patients with follicular carcinoma, elevated Tg levels, and positive RAI uptake. At the lesion level, SSTR-positive lesions were larger, often involved bone metastases, and showed positive ^131^I accumulation.

Somatostatin is a cyclic peptide hormone with complex endocrine, paracrine, and modulatory roles in various organ systems. It inhibits the secretion of hormones and enzymes, regulates cell proliferation, acts as a neurotransmitter, and serves as an immunomodulator [10, 17, 18]. SSTRs belong to a family of seven-subunit transmembrane G protein-coupled receptors comprising five human subtypes: SSTR1–5 [19]. Previous reports have shown higher SSTR2 and SSTR5 expression in all thyroid cancer types than in normal thyroid tissue, based on immunohistochemical analyses of surgical specimens [20, 21]. Despite their presence in thyroid cancer tissue, SSTRs have also been observed in non-tumor stromal cells and peritumor veins [22]. In SPECT imaging analyses, previous studies [23–25] have reported positive SSTR imaging in 19–100% of patients, with positivity defined as uptake above background in DTC. In a pathological semi-quantitative evaluation of all blocks of excised specimens of primary DTC, SSTR was positive in approximately 40% of both papillary and follicular carcinomas [26]. In our cohort, 28.6% (4/14) of cases were SSTR-positive overall. However, when considering each histological type, 9.1% (1/11) of papillary carcinoma cases and 100% (3/3) of follicular carcinoma were SSTR-positive. Few studies have evaluated differences in SSTR expression or SSTR imaging between papillary and follicular carcinomas. Previous studies [27, 28] evaluating SSTR scintigraphy reported similar positivity rates in both RAI-negative and RAI-positive patients, regardless of whether the cancer was papillary or follicular. The higher SSTR positivity observed in follicular carcinoma compared with papillary carcinoma might be related to differences in differentiation status and tumor biology. Follicular carcinoma generally retains more differentiated thyroid features, including the expression of proteins involved in hormone synthesis and SSTRs, whereas papillary carcinoma—particularly BRAF-mutated subtypes—often shows reduced expression of differentiation markers such as NIS and Tg, along with a shift toward enhanced glycolytic metabolism [29]. This biological divergence is consistent with reports that *RAS*-mutated differentiated thyroid carcinomas (more common in follicular carcinoma) maintain differentiated follicular cell features, whereas *BRAF*-driven papillary carcinomas exhibit downregulation of differentiation markers [30]. Therefore, the present findings may reflect the preservation of a more differentiated phenotype and SSTR expression profile in follicular carcinoma, which could also explain its greater concordance between SSTR and RAI uptake. From another perspective, we adopted KS ≥ 2 as the positive criterion, consistent with that used for determining the applicability of PRRT for NETs. This approach evaluates the usefulness of SSTR imaging as a companion diagnostic tool rather than as a diagnostic agent, as previously reported, which might explain the lower positivity rate or pathological differences observed. Moreover, the median size in our study was 16.9 mm, with a bias-controlled group showing a median size of 20 mm, suggesting that the spatial resolution of SPECT might have led to underestimation of tracer accumulation. PET imaging, including Gallium-68 (^68^Ga) PET/CT, provides better spatial resolution than planar scintigraphy or SPECT, enabling the detection of smaller lesions and more precise localization of disease. ^68^Ga-based SSTR imaging has a clinical utility in RAIR DTC, offering valuable insights into the detection of skeletal and lymph node metastases [31]. SSTR-PET also provides a more accurate assessment of the potential utilization of ^177^Lu-DOTATATE therapy; however, this approach has not yet been approved for use in Japan. In addition to technical factors such as spatial resolution, lesion detectability in SSTR imaging is affected by the pharmacologic properties of the tracers. Reubi et al. and Lamberts et al. demonstrated that somatostatin analogues exhibit distinct affinities toward SSTR subtypes (SSTR1–SSTR5), with octreotide and its derivatives showing particularly high affinity for SSTR2 and, to a lesser extent, SSTR5 [32, 33]. These receptor-binding profiles and corresponding IC₅₀ values can influence the visualization of lesions, particularly when tumor cells express mixed or non-SSTR2-dominant subtypes. Therefore, differences in tracer affinity may partly explain the variability in SSTR detectability observed in our study.

There is no clear consensus about the relationship between SSTR expression for lesion detection and ^131^I uptake [24, 25, 34, 35]. Görges et al. demonstrated a slightly higher tumor detection rate with ^111^In-pentetreotide in patients with clearly positive RAI scintigraphy (83% vs. 66%); however, this difference was not statistically significant [25]. SSTR scintigraphy nonetheless showed potential for targeted use in ^131^I-negative cases. Middendorp et al. [36] evaluated 12 ^131^I-positive and ^131^I-negative patients with recurrent DTC, totaling 104 lesions, using ^68^Ga-DOTATOC PET/CT, and found 80.6% (25/31) of ^131^I-positive patients. However, 35.6% (26/73) were detected only in ^131^I-negative patients. In our cohort, there was only one SSTR-positive case (representative case 2, Fig. 2) among the 10 ^131^I-negative cases. These findings suggest that SSTR expression may be increased in lesions with moderate loss of differentiation, but may be lost in those with severe differentiation loss.

In patients with DTC, a reciprocal pattern of iodine and ^18^F-FDG uptake, known as the “flip-flop relationship,” has been confirmed [37]. In our study, ^18^F-FDG SUVmax showed a moderate correlation with SSTR SUVmax but was not an independent predictor of SSTR positivity. SSTR expression has been reported to be independent of glucose transporter overexpression [31]. SSTR and ^18^F-FDG are thereby considered complementary to each other, as 33% of cases were positive for SSTR even when ^18^F-FDG was negative [38]. This may be due to attributable differences in the DTC subtypes studied, highlighting the need for more comprehensive research. This study found that SSTR positivity correlated with elevated Tg levels, consistent with previous reports [25, 38]. Higher Tg levels are thought to enhance tumor detectability in SSTR imaging. Additionally, the high frequency of SSTR positivity in bone lesions was consistent with previous reports [31, 35]. Previous studies of NETs have reported that the tumor microenvironment of metastatic sites can influence SSTR expression and that expression may differ between primary and metastatic lesions, showing increased heterogeneity [39–41]. In cases of thyroid carcinoma, a similar mechanism may exist, whereby the bone microenvironment enhances SSTR expression in osseous metastases, whereas non-osseous lesions exhibit lower expression due to different local conditions. Furthermore, the degree of tumor differentiation may also modulate these interactions. Further studies are needed to clarify these underlying mechanisms.

The clinical application of therapeutic radiopharmaceuticals for SSTR-positive thyroid cancer has advanced significantly. Maghsoomi et al. conducted a systematic review demonstrating the efficacy and safety of PRRT in patients with RAIR-DTC. Biochemical and objective responses were observed in 25.3 and 10.5% of patients, respectively, among a total of 157 cases [13]. In patients treated with ^177^Lu-DOTATATE, only mild and transient haematological or renal complications have been reported. Versari et al. used ^68^Ga-DOTATOC PET/CT to identify patients with RAIR-DTC for PRRT and reported that 64% of treated patients achieved partial response or stable disease [42]. However, their positivity criteria differed from those used in our study, as they defined any uptake above background as positive. In NETs, lesions demonstrating uptake in the liver or higher are considered eligible for treatment. In this study, cases that met this criterion were regarded as positive. It has been reported that high or low accumulation of ^68^Ga-DOTATOCs is not associated with therapeutic efficacy in patients with DTC [43]. However, these findings are based on a small number of cases, and future follow-up studies are warranted. The results of our study will be valuable for identifying suitable candidates for future studies. PRRT offers several potential advantages, including the avoidance of iodine restriction, shortened isolation periods, and prevention of salivary gland damage. Additionally, due to the shortage of radiotherapy rooms in Japan, “special measures for patient rooms” have been approved for PRRT [44], which may allow for more efficient use of treatment resources. In the future, a transition to alpha-particle therapy may be considered, and its application in patients with DTC is anticipated.

In addition to differences in positivity criteria and pathology, some of the disparities observed may be due to racial factors or genetic variations. *BRAF* mutations are the most common genetic alterations in papillary carcinoma, detected in 60–80% of adult Japanese patients, which is higher than rates reported in Europe and the United States (40–60%) [45]. *BRAF* mutations suppress thyroid cell-specific gene expression and lead to the depletion and dedifferentiation of NIS, which are thought to be involved in resistance to RAI [46]. Reports have also linked *BRAF* mutations to tumor grade and prognosis in papillary carcinoma, including extrathyroidal invasion, lymph node metastasis, progression, and recurrence. However, studies of Japanese patients with papillary carcinoma have not demonstrated a significant association between *BRAF* mutations and prognosis [47]. RAS point mutations occur early in tumor development and are detected in both follicular adenomas and follicular carcinomas. Additionally, RAS mutations occur in 30–50% of follicular carcinomas and are more frequent than in follicular adenomas [48]. However, to the best of our knowledge, no studies have investigated the relationship between these genetic alterations and SSTR expression.

This study has some limitations. Although it is a prospective investigation, the major limitation of this study is the small sample size, which restricts the strength of the conclusions. Accordingly, the correlations observed between SSTR expression and clinical features should be considered as exploratory and hypothesis-generating. Larger, multicenter studies are required to validate these findings and clarify their clinical significance. In particular, since no cases of follicular carcinoma without ^131^I accumulation were included, whether all follicular carcinomas demonstrated high SSTR expression remains unclear. Although ROC analysis identified potential lesion-level thresholds for predicting SSTR positivity, these results should be interpreted with caution because they were derived from a relatively small dataset. Nonetheless, the 2,000 bootstrap replications effectively captured the inherent variability and uncertainty of the optimal cutoff. Theoretically, positive RAI uptake might indicate a higher likelihood of SSTR expression and potential eligibility for PRRT; however, one patient in our cohort showed negative RAI uptake but positive SSTR finding. Therefore, the thresholds identified in this study are unlikely to be directly applicable in clinical practice. Furthermore, since molecular and genetic factors influencing SSTR expression were not assessed, the discussion of threshold-based clinical implications was intentionally limited. A previous report concluded that thyroid hormone suppression therapy does not need to be discontinued to perform SSTR imaging, suggesting that TSH stimulation has little effect on SSTR expression [24]. In this study, all patients were on a low-iodine diet; however, the effect of this diet on SSTR expression remains unclear. Additionally, genetic mutations associated with thyroid cancer were not examined in this study. The Deauville score was used to qualitatively assess ^18^F-FDG uptake. Although this scoring system was originally validated for lymphoma, it has also been applied in studies of thyroid nodules and other malignancies [49, 50]. Although its suitability for DTC remains uncertain, the Deauville score provides a reproducible and standardized qualitative assessment, which was considered appropriate for the present analysis. Another limitation pertains to the timing of the ^18^F-FDG PET/CT studies, which were performed within a 180-day window. This introduces the potential for timing bias, as ^18^F-FDG uptake may not accurately reflect the metabolic status of the disease at the exact time of SSTR and ^131^I imaging.

In conclusion, this is the first study to comprehensively evaluate high SSTR expression in Japanese patients with DTC. Our findings indicated that patients with follicular thyroid carcinoma, elevated Tg levels, larger tumor size, and positive ^131^I uptake are more likely to have SSTR-positive lesions. These results may contribute to the development of novel therapeutic strategies using radiopharmaceuticals targeting SSTR in DTC.

## Supplementary Information

Below is the link to the electronic supplementary material.

Supplementary Material 1
