# Supplementary material for: Somatostatin receptor expression for peptide receptor radionuclide therapy in Japanese patients with recurrent or metastatic differentiated thyroid cancer
Source: Ann Nucl Med. 2026 Jan 23;40(5):539–51. doi: 10.1007/s12149-026-02155-7 (PMC13124881; doi:10.1007/s12149-026-02155-7)
Supplement: Supplementary file 1 — Supplementary Material 1 [file 12149_2026_2155_MOESM1_ESM.docx]

Supplementary Table S1. Lesion size and SUVmax by organ for all evaluable lesions

| **Organ** | **Number of lesions** | **Lesion size (mm), median (range)** | **FDG SUVmax, median (range)** | **^131^I SUVmax, median (range)** | | **SSTR SUVmax, median (range)** | | **Interpretation** | |
| --- | --- | --- | --- | --- | --- | --- | --- | --- | --- |
| Lymph node | 9 | 22 (10–40) | 3.1 (0.8–9.0) | 5.5 (0.2–12.0) | 0.8 (0.1–2.0) | | Lymph node lesions tended to show higher RAI and SSTR uptake. | |  |
| Lung | 3 | 15 (8–30) | 2.0 (0.5–6.8) | 2.3 (0.0–8.5) | 0.4 (0.1–1.1) | | Lung lesions generally showed lower SSTR expression. | |  |
| Bone | 17 | 40 (20–60) | 6.0 (1.2–10.0) | 7.5 (3.0–11.0) | 1.2 (0.6–2.5) | | Bone metastases were larger and had high SSTR and RAI avidity. | |  |
| *FDG* fluorodeoxyglucose, *SSTR* somatostatin receptor, *SUV* standardized uptake value | | | | |  | |  | |  |

Supplementary Table S2. Lesion size and SUVmax by organ for representative lesions

| **Organ** | **Number of lesions** | | **Lesion size (mm), median (range)** | | **FDG SUVmax, median (range)** | | **^131^I SUVmax, median (range)** | | | | **SSTR SUVmax, median (range)** | | | **Interpretation** | | |
| --- | --- | --- | --- | --- | --- | --- | --- | --- | --- | --- | --- | --- | --- | --- | --- | --- |
| Lymph nodes | | 7 | | 22 (10–40) | | 3.1 (0.8–9.0) | | 5.5 (0.2–12.0) | | 0.8 (0.1–2.0) | | | Lymph node lesions exhibited moderate to high RAI and SSTR uptake. | | |  |
| Lung | | 3 | | 15 (8–30) | | 2.0 (0.5–6.8) | | 2.3 (0.0–8.5) | | 0.4 (0.1–1.1) | | | Lung lesions tended to have lower SSTR expression compared with other sites. | | |  |
| Bone | | 7 | | 40 (20–60) | | 6.0 (1.2–10.0) | | 7.5 (3.0–11.0) | | 1.2 (0.6–2.5) | | | Bone metastases were generally larger and demonstrated high RAI and SSTR avidity. | | |  |
| *FDG* fluorodeoxyglucose, *SSTR* somatostatin receptor, *SUV* standardized uptake value | | | | | | | | |  | | |  | | |  |  |
